# Editorial: Management of spine pathologies in geriatric patients

**DOI:** 10.3389/fmed.2025.1688766

**Published:** 2025-09-12

**Authors:** Ismail Zaed, Gabriele Capo, Aria Nouri, Luca Ricciardi

**Affiliations:** ^1^Department of Neurosurgery, Neurocenter of South Switzerland, Ente Ospedaliero Cantonale, Lugano, Switzerland; ^2^Department of NESMOS, Sapienza University, Rome, Italy; ^3^Department of Neurosurgery, IRCCS Humanitas Research Hospital, Milan, Italy; ^4^Division of Neurosurgery, Department of Clinical Neurosciences, Geneva University Hospitals, Geneva, Switzerland; ^5^UOC di Neurochirurgia, Azienda Ospedaliera Universitaria Sant'Andrea, Rome, Italy; ^6^Dipartimento NESMOS, Sapienza Università di Roma, Rome, Italy

**Keywords:** spine, geriatric, osteoporosis, fracture, degenerative spine

Population aging is reshaping spine care. Older adults often present with a dense tangle of multimorbidity, frailty, osteoporosis, sarcopenia, polypharmacy, and goals of care that prioritize function and independence as much as pain relief (1, 2). This Research Topic set out to synthesize pragmatic, patient-centered advances across the continuum—from diagnosis and anesthesia to intervention, complication surveillance, and rehabilitation—to help clinicians individualize decisions for geriatric patients with spine disease.

The eight contributions collected here move the field forward in four overarching ways: (1) sharpening risk prediction, (2) elevating peri-procedural safety, and (3) testing realistic rehabilitative and non-pharmacologic strategies, while also spotlighting emerging (4) diagnostic tools.

1) Sharpening risk prediction in osteoporotic vertebral compression fractures (OVCF).

Two original studies offered complementary perspectives on why some older patients sustain *new* vertebral compression fractures (NVCFs) after vertebral augmentation, and how to anticipate that risk in a clinical setting. A large single-center study (n=420) developed and internally validated a streamlined nomogram in which cement leakage, poor cement dispersion, and pre-existing endplate fractures were independent predictors of NVCFs, demonstrating very strong discrimination (training AUC 0.974; validation AUC 0.965). The model is simple enough to implement at the point of care and focuses attention on modifiable intraoperative quality targets (e.g., cement handling and dispersion) (Gao et al.). A second, two-cohort analysis (internal *n* = 235; external *n* = 105) integrated paraspinal muscle health into risk stratification. It showed that the multifidus skeletal muscle index (SMI)—a surrogate of sarcopenia—along with surgical approach and spinal CT values, predicted NVCFs; the resulting nomogram demonstrated reasonable performance across validations and, notably, greater negative predictive value, which is useful for reassuring low-risk patients. The message is clear: bone and muscle form a functional unit in the aging spine, and sarcopenia matters when counseling about augmentation (Tang et al.).

Together, these studies support pre- and intra-operative checklists that couple imaging of paraspinal muscle quality with cement technique standards. They also justify post-procedure secondary prevention that addresses both osteoporosis and sarcopenia.

2) Improving safety around interventions.

Minimally invasive augmentation is effective for selected OVCFs, but geriatric anesthesia and rare complications remain important considerations. In a randomized comparison of sedative regimens during percutaneous kyphoplasty, ciprofol (a propofol analog) was found to provide more stable hemodynamics and less respiratory depression than traditional propofol while achieving comparable sedation—an attractive profile for older adults with limited cardiopulmonary reserve (Yao et al.). At the other end of the safety spectrum, a vivid case report described a patient who experienced simultaneous spinal subdural and epidural hematomas after percutaneous kyphoplasty (PKP), reminding us that even “routine” procedures can produce rare, catastrophic bleeds. The clinical pearl is vigilance: prompt recognition of disproportionate pain or a new neurological deficit post-PKP should trigger urgent imaging and decompression when indicated, with careful attention to coagulation status during the preoperative workup (Tang et al.). Finally, this Research Topic included the protocol for a randomized controlled trial directly comparing percutaneous vertebroplasty vs. optimized conservative care in OVCFs, an area where practice varies. By specifying outcomes a priori and enrolling a geriatric cohort, the trial is poised to clarify effect sizes that matter to older patients—pain relief trajectories, mobility, and downstream fracture risk (Gao et al.).

3) Real-world rehabilitation and non-pharmacologic options.

Functional recovery and preservation of quality of life are generally the main aims in the management of geriatric patients. In a pragmatic inpatient study of older adults with degenerative spinal disease, robot-assisted gait training did outperform conventional therapy in terms of walking distance and satisfaction over a short, two-week horizon; however, it was most helpful for those with severe baseline mobility limitations, suggesting a role as a targeted adjunct rather than a universal solution. For resource-constrained systems, this nuance matter (Jee et al.). Complementing this, a multicenter randomized placebo-controlled trial protocol tested the effectiveness of acupuncture in treating degenerative lumbar spinal stenosis, an archetypal geriatric condition where pharmacologic options are limited and surgery is not always preferable. Positive data about the effectiveness and safety of optimal acupuncture therapy could expand low-risk, scalable treatments that align with older patients' preferences (Shi et al.).

4) Emerging diagnostics for smarter decisions.

Beyond structure, metabolism matters. A systematic review and meta-analysis highlighted proton MR spectroscopy in cervical spondylotic myelopathy (CSM), linking metabolite ratios to clinical severity and functional outcomes. Although currently limited by small samples and technical variability, MR spectroscopy shows promise as a non-invasive biomarker for refining the timing of surgery and personalizing follow-up. Larger, standardized studies are warranted (Montes-González et al.).

Where do these studies leave us? In our opinion, there are three different practical takeaways from these articles:

1. Think “bone–muscle” when planning augmentation. Incorporate paraspinal muscle assessment alongside bone health and prioritize cement technique. Use simple nomograms to flag risk factors early—and to guide conversations about expectations and prevention (Tang et al.).
2. Match intervention intensity to physiologic reserve. Favor hemodynamically gentle sedation strategies for PKP in frail patients, and maintain a low threshold for investigating atypical pain or neurologic changes in rare hematomas. Protocolized trials comparing augmentation to high-quality conservative care will help determine appropriate indications (Chen et al.; Deng and Liu).
3. Prioritize function with scalable support. Robot-assisted training may be most valuable for those who are most impaired; meanwhile, rigorous trials of acupuncture may broaden low-risk options for lumbar stenosis (Jee et al.).

## Future directions

The common theme throughout this Research Topic is personalization—using better predictors, gentler anesthetic choices, vigilant monitoring of complications, and realistic rehabilitation plans to address the needs of older adults. Future work should emphasize multicenter external validation of risk tools (including muscle metrics), standardized safety protocols for augmentation procedures, and patient-reported outcomes that capture mobility and independence (3, 4). The promise of metabolic imaging in CSM is real, and investment in technical standardization could transform it from a research tool to a bedside biomarker.
